# Efficacy and safety of Guizhi-Shaoyao-Zhimu decoction in the treatment of rheumatoid arthritis: A protocol for systematic review and meta-analysis

**DOI:** 10.1097/MD.0000000000024416

**Published:** 2021-03-05

**Authors:** Jing Ye, Renliang Li, Ziyi Hu, Ping Zhang, Liangji Liu

**Affiliations:** a Jiangxi University of Traditional Chinese Medicine; b The Affiliated Hospital of Jiangxi University of Traditional Chinese Medicine, Nanchang, Jiangxi Province, P.R. China.

**Keywords:** Guizhi-Shaoyao-Zhimu decoction, protocol, rheumatoid arthritis, systematic review

## Abstract

**Background:**

Rheumatoid arthritis (RA) is a significant public health problem associated with a substantial burden of functional disability. The Guizhi-Shaoyao-Zhimu decoction (GSZD), a traditional medicine, has been used in China for a long time to treat RA. This study aimed to systematically investigate the efficacy and safety of GSZD in the treatment of RA.

**Methods:**

We will search the electronic databases of PubMed, Embase, the Cochrane Library, China National Knowledge Infrastructure, the Chongqing VIP Chinese Science and Technology Periodical Database, Wanfang Database, and China Biomedical Literature Database, and also manually search the Chinese Clinical Trial Register and unpublished studies or references, with the establishment up to February 2021. According to the inclusion and exclusion criteria, we will screen the literature, and the data are extracted independently by the 2 researchers. We will collect RCTs of GSZD in the treatment of RA. RevMan5.3 will be used for statistical analysis. According to the Grades of Recommendation, Assessment, Development, and Evaluation (GRADE), we will appraise each outcome quality evidence.

**Results:**

We will publish the results in a peer-reviewed journal.

**Conclusion:**

We will evaluate the efficacy and safety of GSZD in treating RA.

**Unique INPLASY number:**

INPLASY2020120147.

## 1 Introduction

Rheumatoid arthritis (RA) is a chronic autoimmune disease characterized by persistent synovitis, progressive joint injury, deformity, and even disability.^[1]^ The global prevalence of RA was 460 per 100,000 population.^[2]^ Fatigue, joint inflammation, and deformity are the main complications of rheumatoid arthritis, which can damage physical function, work efficiency, and activities of daily life, as well as overall emotional health.^[3]^ RA is associated with the enormous economic burden of individual patients, their families, and society.^[4]^ It is estimated that the total annual financial burden in Europe and the United States is 45.3 billion euros and 41.6 billion euros, respectively.^[5]^ RA is considered a multifactorial disease, where various genetic and environmental factors.^[6,7]^

The main goal of RA treatment is to relieve the symptoms and physique in the active stage of inflammation.^[8]^ Drugs used to treat RA include non-steroidal anti-inflammatory drugs (NSAIDs), corticosteroids, disease-modifying arthritis drugs (DMARDs).^[1]^ NSAIDs can reduce inflammation and pain by inhibiting cyclooxygenase and reducing prostaglandin synthesis.^[9]^ However, many of the adverse effects of NSAIDs are also related to inhibition of prostaglandin production, making their use problematic in some patient populations.^[10]^ Corticosteroids can relieve clinical symptoms, but it can cause adverse reactions of water and salt metabolism, sugar, fat, and protein metabolism disorder, and severe infection when used for a long time. DMARDs can improve patients’ symptoms, erythrocyte sedimentation rate, and delay joint bone destruction if it can be used early.^[11]^ However, such drugs often have various toxic and side effects.

In China, natural products have been used throughout recorded history and are still in use for RA and its symptoms.^[12]^ RA belongs to the “Bi Zheng” category of traditional Chinese medicine (TCM). Guizhi-Shaoyao-Zhimu (GSZD) is the first choice for clinical treatment of RA in Chinese traditional medicine, which originated from *JingGuiYaoLue.* Zhang et al^[13]^ found that GSZD has an anti-rheumatic effect on collagen-induced arthritis rats, and its possible mechanism is related to inhibition of inflammatory response, inhibition of invasion and migration of synovium fibroblasts, and induction of apoptosis of synovium fibroblasts. Guo et al^[14]^ also discovered that GSZD may partially attenuate RA by reversing inflammation-immune system imbalance and regulating the HDAC1-HSP90AA1-NFKB2-IKBKB-TNF-α signaling axis.

There are currently 8 systematic reviews of GSZD in the treatment of rheumatoid arthritis, 7 of which were published in Chinese,^[15–21]^ and 1 was published in English.^[22]^ However, there are also some defects, such as insufficient retrieval, unregistered, few result indicators, inadequate evaluation of evidence quality, long publication time, and so on. Meta-analysis is one of the highest levels of evidence in evidence-based research. Therefore, there is an urgent need to update and improve the meta-analysis of GSZD for RA. In this study, we aimed to evaluate the efficacy and safety of GSZD for RA objectively and provide reliable evidence for the clinical application of GSZD in RA.

## 2 Methods

### 2.1 Protocol and registration

This protocol has been registered on the INPLASY website, and the registration number is INPLASY2020120147 (URL https://inplasy.com/inplasy-2020-12-0147/). This report will be performed by the Preferred Reporting Items for Systematic Review and Meta-Analysis Protocols (PRISMA-P).^[23]^

### 2.2 Data source

#### 2.2.1 Electronic search

We will search the following databases from the establishment to February 2021: PubMed, Embase, the Cochrane Library, China National Knowledge Infrastructure, the Chongqing VIP Chinese Science and Technology Periodical Database, Wanfang Database, and China Biomedical Literature Database. The PubMed strategy details are shown in Table 1, and the Chinese databases will use these items translated by Chinese.

**Table 1:** PubMed search strategy.

Number	Search terms
#1	Arthritis, Rheumatoid [MeSH]
#2	Rheumatoid Arthritis [title/abstract]
#3	#1 OR #2
#4	Guizhi-Shaoyao-Zhimu decoction [title/abstract]
#5	GSZD [title/abstract]
#6	Drugs, Chinese Herbal [MeSH]
#7	Chinese Drugs, Plant [title/abstract]
#8	Chinese Herbal Drugs [title/abstract]
#9	Herbal Drugs, Chinese [title/abstract]
#10	Plant Extracts, Chinese [title/abstract]
#11	Chinese Plant Extracts [title/abstract]
#12	Extracts, Chinese Plant [title/abstract]
#13	#4 OR #5 OR #6 OR #7 OR #8 OR #9 OR #10 OR #11 OR #12
#14	randomized controlled trial [Publication Type]
#15	randomised [Title/Abstract]
#16	placebo [Title/Abstract]
#17	#14 OR #15 OR #16
#18	#3AND #13 AND #17

#### 2.2.2 Other sources of search

We will also manually search the Chinese Clinical Trial Register and unpublished studies or references. The manual review of references in published articles will be conducted to identify other relevant studies.

### 2.3 Inclusion criteria

#### 2.3.1 Type of studies

Randomized controlled trials (RCTs) will be included in this review, regardless of whether the blind method and allocation concealment are used.

#### 2.3.2 Type of participants

Patients who met the standard of RA diagnosis will be included.

#### 2.3.3 Type of interventions

The treatment group was treated alone with GSZD, or GSZD combined with western medicine.

#### 2.3.4 Type of comparators

The control group was treated with western medicine without GSZD.

#### 2.3.5 Types of outcome measures

##### 2.3.5.1 Primary Outcome

The total effective rate and visual analogue scale (VAS) score.

##### 2.3.5.2 Secondary outcomes

(1) Swollen joint count (SJC);
(2) Morning stiffness time;
(3) Inflammatory indicators (such as CRP and ESR);
(4) Rheumatoid factor (RF);
(5) Incidence of adverse events.

### 2.4 Exclusion criteria

(1) Republished literature;
(2) Research on insufficient data or lack of access to the full text;
(3) Case report, reviews, basic research, nonRCT.

### 2.5 Studies selection

**Figure 1:**
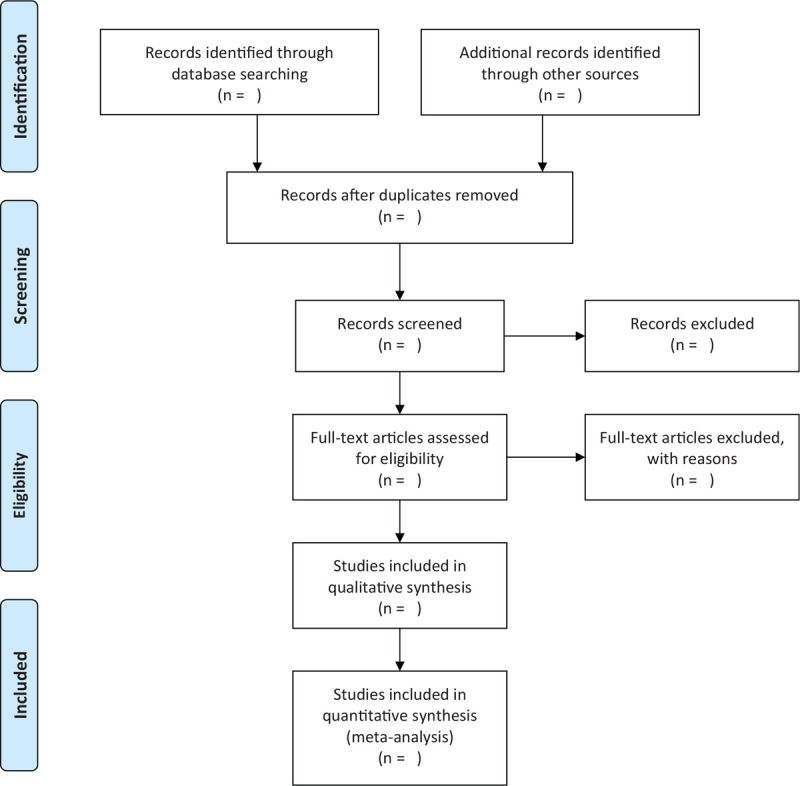
Flow diagram of literature retrieval.

We will eliminate duplicate studies from the search results by Endnote X9 software (Camelot UK Bidco Limited (Clarivate) on network). Two reviewers will screen the literature independently. Provided that the 2 reviewers have different opinions, whether or not the literature should be included, they should resolve it by discussion. The selection will be performed according to the PRISMA flow chart shown in Fig. 1.

### 2.6 Data extraction and management

The data will be screened and extracted by 2 researchers. The data extraction content includes the Author's name, Year of publication, Title, Country, The enforcement time of the study, Study design, Sample size, Participants, Intervention, Comparison, Outcome, and some relevant characteristics.

### 2.7 Assessment of the methodological quality

There may be biases in clinical trials from selecting and assigning subjects, implementing interventions, following up matters, and measuring and reporting findings at every stage. Thus, RCTs will be evaluated through the bias risk assessment tool (Cochrane Handbook for Systematic Reviews of Interventions).^[24]^ It includes the following 6 items: random sequence generation; allocation concealment; blinding of participants, caregivers, outcome assessors; incomplete outcome data; selective outcome reporting; and other bias. According to each study's results, the 2 researchers made “low-risk,” “high-risk,” or “unclear risk” assessment of the above 6 items, independently. If the 2 researchers occur different opinions, the objection will be decided by the third reviewer.

### 2.8 Measures of treatment effect

The risk ratio (RR) and its 95% confidence intervals (CIs) will be used in the dichotomous variables. Continuous variables will be statistically analyzed using weighted mean difference (WMD) or standardized mean difference (SMD) and its 95% CIs.

### 2.9 Dealing with missing data

We will first communicate with the corresponding author to get complete data. If we cannot get the missing data, we will do a meta-analysis based on the existing data. If the lost data have no potential impact on our research, we will rule it out.

### 2.10 Assessment of heterogeneity

Heterogeneity will be assessed using a Chi-square test and *I*^*2*^ statistics (*P-*value < .10 or *I*^*2*^ over 50% were defined as substantial heterogeneity).

### 2.11 Data synthesis

RevMan5.3 software (Cochrane collaborate on network) will conduct this meta-analysis. A random-effects model will be used to estimate the pooled primary and secondary outcomes. The forest plots will display the results of the meta-analysis. If the products are not suitable for meta-analysis, we will conduct a descriptive analysis. Only when >10 RCTs are included can we use funnel charts to assess publication bias.

### 2.12 Sensitivity analysis

We will use the leave-one-out method for sensitivity analysis to judge the stability of outcome indicators.

### 2.13 Subgroup analysis

According to the treatment group with GSZD alone or combined with Western medicine, we will conduct a separate meta-analysis. We will carry out a subgroup analysis according to different dosage forms of GSZD, Chinese herbal compound, and extra western medicine in the control group.

### 2.14 Summary of evidence

According to the Grades of Recommendation, Assessment, Development, and Evaluation (GRADE), each outcome quality evidence will be appraised.^[25]^

### 2.15 Ethics and dissemination

Ethical approval does not apply to this study, as no individual data from participants. We will publish this systematic review in a peer-reviewed journal.

## 3 Discussion

According to the theory of TCM, the incidence of RA is due to the patients’ body deficiency, overstrain, invasion by wind, cold and dampness, evil qi entering the meridians and collaterals, leaving in the joints, blocking the movement of qi and blood, causing joint flexion and extension, pain, numbness, and other pathological reactions. Besides, the evil qi stays in the body, causing damage to the bones, resulting in its deformation and dysfunction. Guizhi-Shaoyao-Zhimu decoction (GSZD) is a classic herbal preparation from *JingGuiYaoLue.* It is composed of 9 herbs, including Guizhi (Ramulus Cinnamomi), Baishao (Radix Paeoniae Alba), Zhimu (Anemarrhenae Rhizoma), Fangfeng (Saposhnikoviae Radix), Baizhu (Atractylodes Macrocephala Koidz), Fuzi (Aconiti Lateralis Radix Praeparata), Mahuang (Ephedra Herba), Shengjiang (Zingiber Officinale Roscoe), and Gaocao (licorice). According to the theory of TCM, GSZD can “disperse wind and cold, nourish yin and clear heat.” Previous studies have proved that GSZD has good efficacy and safety in RA treatment.^[22]^ In recent years, there has been a significant increase in the number of high-quality RCT about GSZD in RA treatment, so it is necessary to update this meta-analysis. We hope that this study provides a comprehensive review of current evidence for the effectiveness and safety of GSZD in RA treatment and guide clinical decision-making.

## Author contributions

**Conceptualization:** Jing Ye and Ziyi Hu.

**Data curation:** Renliang Li and Ping Zhang.

**Methodology:** Renliang Li, Ping Zhang, Liangji Liu, and Ziyi Hu.

**Software:** Ping Zhang.

**Supervision:** Ziyi Hu and Liangji Liu.

**Writing – original draft:** Renliang Li, Ping Zhang, and Jing Ye.

**Writing – review & editing:** Jing Ye, Ziyi Hu, and Liangji Liu.
